# Graphene-Based Aerogels for Biomedical Application

**DOI:** 10.3390/gels9120967

**Published:** 2023-12-09

**Authors:** Yeongsang Kim, Rajkumar Patel, Chandrashekhar V. Kulkarni, Madhumita Patel

**Affiliations:** 1 Bio-Convergence, Integrated Science and Engineering Division (ISED), Underwood International College, Yonsei University, 85 Songdogwahak-ro, Yeonsugu, Incheon 21938, Republic of Korea; 2 Energy & Environmental Science and Engineering (EESE), Integrated Science and Engineering Division (ISED), Underwood International College, Yonsei University, 85 Songdogwahak-ro, Yeonsugu, Incheon 21938, Republic of Korea; rajkumar@yonsei.ac.kr; 3 Centre for Smart Materials, School of Pharmacy and Biomedical Sciences, University of Central Lancashire, Preston PR1 2HE, UK; cvkulkarni@uclan.ac.uk; 4 Department of Chemistry and Nanoscience, Ewha Womans University, 52 Ewhayeodae-gil, Seodaemun-gu, Seoul 03760, Republic of Korea

**Keywords:** graphene aerogel, wound healing, hemostasis, bilirubin adsorption, drug delivery, biosensor

## Abstract

Aerogels are three-dimensional solid networks with incredibly low densities, high porosity, and large specific surface areas. These aerogels have both nanoscale and macroscopic interior structures. Combined with graphene, the aerogels show improved mechanical strength, electrical conductivity, surface area, and adsorption capacity, making them ideal for various biomedical applications. The graphene aerogel has a high drug-loading capacity due to its large surface area, and the porous structure enables controlled drug release over time. The presence of graphene makes it a suitable material for wound dressings, blood coagulation, and bilirubin adsorption. Additionally, graphene’s conductivity can help in the electrical stimulation of cells for improved tissue regeneration, and it is also appropriate for biosensors. In this review, we discuss the preparation and advantages of graphene-based aerogels in wound dressings, drug delivery systems, bone regeneration, and biosensors.

## 1. Introduction

Aerogels are solid polymeric networks that are incredibly light and exhibit 95% porosity. It is generally prepared using the sol–gel method, which involves converting a liquid (sol) into a gel phase. In 1931, Samuel Stephens Kistler first prepared silica aerogels by critical point drying [1]. Later, researchers explored several aerogels, such as synthetic aerogels from polyurethanes, polyurethane-acrylate, polystyrene, natural aerogels from cellulose, alginate starch, etc. [2].

Graphene-based aerogel (GA) has recently garnered significant attention due to its high pore volume, conductivity, and environmentally friendly nature [3,4]. Graphene is a single-layer graphite generally synthesized by Hummer’s method [5]. The remarkable physical and chemical properties of graphene and its distinct two-dimensional structure open up a wide range of possible uses in nanoelectronics, energy technology, and sensors [6]. Beyond this, graphene has attracted interest in biomedicine, including bioimaging, phototherapy, gene delivery, and biosensing [7].

GAs are three-dimensional networks of graphene, where graphene sheets are the dominant building blocks and crosslink by weak physical interactions such as hydrogen bonding, electrostatic interaction, and π–π interactions [3]. These aerogels possess the highly porous structure of aerogels and most of the outstanding properties of graphene. They are typically synthesized by freezing or supercritical drying of the graphene wet gels [8]. Their excellent intrinsic qualities also result in robust mechanical strength and high transfer rates for both mass and electrons [9]. Recently, GA has been widely used in energy storage [10,11], oil/water separation [12,13], electromagnetic shielding [14], methylene blue, heavy metal, dye adsorption [15], and biomedical engineering [7]. GA is a promising material for biomedical applications because its specific microstructure mimics the extracellular matrix environment [7]. Moreover, the direct interaction of GA with biological molecules, cells, and tissues deals with many therapeutic demands. GA forms an effective barrier against environmental elements as a wound dressing. It can absorb exudate, allow gas exchange, and prevent the worsening of microorganisms [16]. The antibacterial properties of GA can inhibit bacterial growth and hinder biofilm formation [17]. The porous, interconnected network mimics the lightweight and flexible structure of the bone [18], and its high reactivity, efficient dispersal in solution, and stability show promise as an efficient drug carrier [19]. Additionally, studies have demonstrated that GA’s substantial specific surface area, high porosity, and complex three-dimensional network topologies can increase the sensitivity of flexible pressure sensors [20].

In this review, we briefly outline the preparation methods and potential biomedical applications of GA. We discuss various techniques to synthesize GA and then explore their potential usage in wound healing, drug delivery, bilirubin absorption, and bone regeneration, as well as biosensors.

## 2. Preparation of Graphene-Based Aerogel

**Figure 1:**
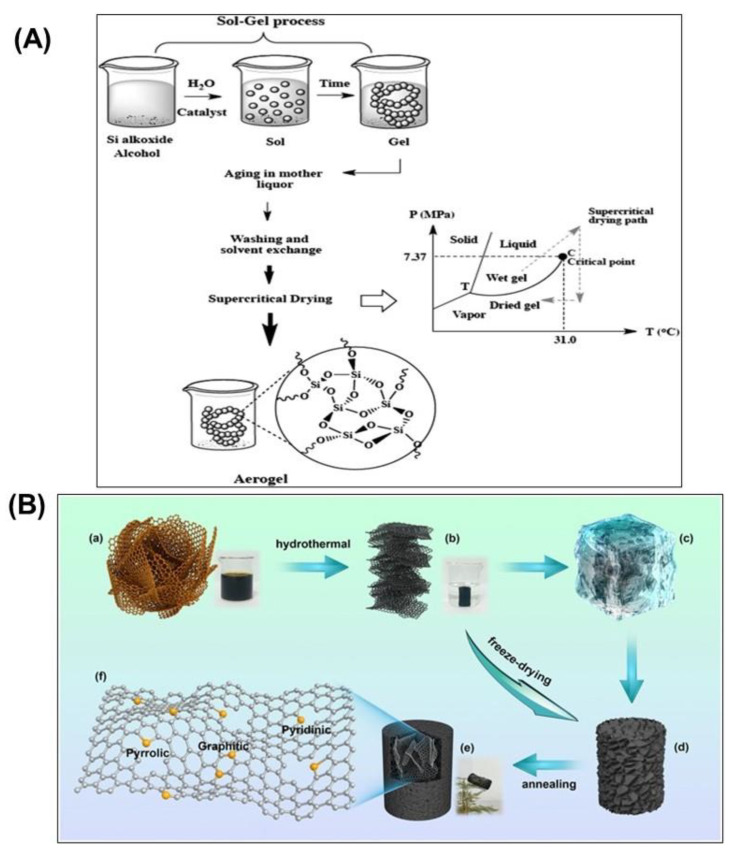
Schematic showing sol–gel (**A**) and hydrothermal synthesis (**B**) of graphene-based aerogels [22,23].

The microstructure of GA strongly affects its properties, such as mechanical properties and thermal conductivities, which can be modified by precisely controlling synthesis conditions [21]. There are three standard methods for preparing GA: the sol–gel method, freeze casting, and hydrothermal reduction [8]. The sol–gel synthesis process and hydrothermal reduction with its detailed steps are presented in Figure 1 [22,23]. GA has great physiological and biological properties, characterized by several techniques. Compressive modulus, water uptake ability, pore size distribution, and surface topography evaluate GA’s physical properties, while cytocompatibility and in vivo degradability are commonly used to study biological properties [24,25]. 

### 2.1. Sol–Gel Method

The simplest method for the synthesis of GA is the sol–gel method. When using graphene sheets as a precursor, their low solubility in almost all solvents makes them difficult to work with. Therefore, the method uses graphene oxide (GO) as an alternative precursor. The abundant oxygenated functional groups, such as epoxides, hydroxyls, and carboxylic acids, in GO allow it to disperse in various solvents [7]. The gelation is conducted by using suspensions of GO sheets above the critical gel concentrations [15]. The suspensions cause the attractive and repulsive forces to be imbalanced between the suspended GO sheets [26]. The oxygenated functional groups in GO provide chemical crosslinking sites that can be eliminated during synthesis. By controlling pH, adding crosslinking agents, or applying ultrasound, a three-dimensional structure can form from the suspension. The majority of extra crosslinking agents are the molecules of bifunctional polymers that attach to and entangle the functional groups on GO [27]. This causes the restoration of structures like graphene, termed reduced graphene oxide (rGO), and hydrogel production [28]. After drying the hydrogel, the GA is synthesized. This method is easy and economical. It can be integrated with other methods, such as reduction or crosslinking. However, there is a limitation to using pure graphite as a precursor due to its low solubility in most solvents. During the sol–gel method, some factors, such as temperature and precursor concentration, can be controlled to modify the final pore size of the aerogel [7]. In Table 1, we summarize the available control variables and their impacts on the pore size of aerogel in the sol–gel method.

### 2.2. Freeze Casting

Freeze casting is a commonly used and straightforward method to synthesize GA. The morphology of the aerogel is modified by ice crystal nucleation and growth [34]. The complex and dynamic interactions between liquid particles and the particles themselves determine the outcome of the synthesizing technique. Functional groups present on GO can form H-bonds with water, which can impact the growth of ice crystals. The temperature gradient affects the arrangement of grown ice crystals between GO sheets. The morphology of the colloids is determined by the direction of ice growth and the crystalline size [7]. After sublimating ice crystals, a three-dimensional, highly porous structure can be synthesized.

In freeze casting, it is easy to control the pore size of aerogel through the temperature gradient [35]. Additionally, it is ecofriendly. However, precise equipment is needed to control the temperature gradient finely [36]. Likewise, during the freeze casting, factors such as temperature and freezing speed can be controlled to modify the pore size of the aerogel [35,37]. The available control variables in the freeze-casting method and their impacts on the pore size of aerogel are summarized in Table 2.

### 2.3. Hydrothermal Reduction

GO is the most common precursor for synthesizing GA for hydrothermal reduction due to its high hydrophilicity and oxygen within its surface functional groups [40]. As the gelation proceeds, most oxygen-containing functional groups are eliminated and transformed by the specific hydrothermal environment of high pressure and high temperature, and rGO is formed [41]. As a result, it is confined in a dense multilayer shell and forms macroscopic voids in the gel [42]. Additionally, the self-assembling of rGO leads to monoliths and three-dimensional graphene-based structure formation [43].

During hydrothermal reduction, a reducing agent is not needed. Therefore, it is environmentally friendly and has a low production cost. However, it is hard to control the aerogel microstructure due to the diversity of factors for synthesizing GA. It also needs higher temperature and pressure than other methods [7]. Table 3 shows the available controlled factors for modifying the pore size of aerogel.

## 3. Graphene Aerogel in Biomedical Applications

GA has gained attention in the biomedical field due to its porous structure, high mechanical strength, and biocompatibility. GA provides structural support to surrounding cells and mimics the natural tissue found in vivo. Extensive studies have focused on using GA for various biomedical applications, including wound healing, bilirubin adsorption, drug delivery, bone regeneration, and biosensors. We summarize a few selected studies on the use of GA for biomedical applications in Table 4.

### 3.1. Applications of Graphene-Based Aerogels in Wound Healing and Hemostat Application

GA, as a wound dressing, is anticipated to create an efficient barrier between the wound and its surroundings by preventing the invasion of microorganisms and absorbing exudate, as well as toxic substances [16]. Additionally, GA has the advantages of hemostatic applications because of its large pore volumes, low density, and excellent structural stability [66]. Hemostasis is the natural and complex response of the body to control bleeding, which involves a combination of blood vessels and platelets to prevent significant blood loss [67].

In a study conducted by Fernández and colleagues, a gelatin–graphene oxide (G-GO) aerogel was synthesized both under acidic and basic conditions to create a positively charged and negatively charged surface. The positively charged aerogels displayed superior structural properties, such as stiffness, porosity, and pore sizes, as they encouraged the formation of hydrogen bonding. On the other hand, negatively charged aerogels exhibited better clotting performance, making them suitable for coagulation and wound dressings. They were also biocompatible and promoted fibroblast proliferation [49]. In a later study, researchers found that G-GO aerogel, synthesized using microwave-assisted reactions, has promising potential for wound healing. By adding proanthocyanidins (PAs), an extract of grape skin, the aerogel increased the negative surface charges. The aerogels were biocompatible with human dermal fibroblast cells and can adhere red blood cells to their surfaces, promoting hemostasis by forming stable fibrin networks. The study revealed that the inclusion of 5% PAs increased the total blood coagulation content by 36.6%, while the inclusion of 10% PAs resulted in a lower increase of 24.5% [50]. The ability of PA-loaded G-GO to absorb blood and to maintain cell viability was enhanced in both in vitro and in vivo studies, which was related to oxidation levels and oxygenated groups of PA [68]. Similar results were found in GO and polyethylene glycol (PEG) aerogels loaded with PA grape seed extract. The aerogel was developed by noncovalent interaction and exhibited a significant increase in negative surface charges, which caused more blood coagulation. The coagulated blood content increased to 84% for GO-PEG/PA aerogels after 30 s of reaction compared to GO-PEG aerogel [51]. 

**Figure 2:**
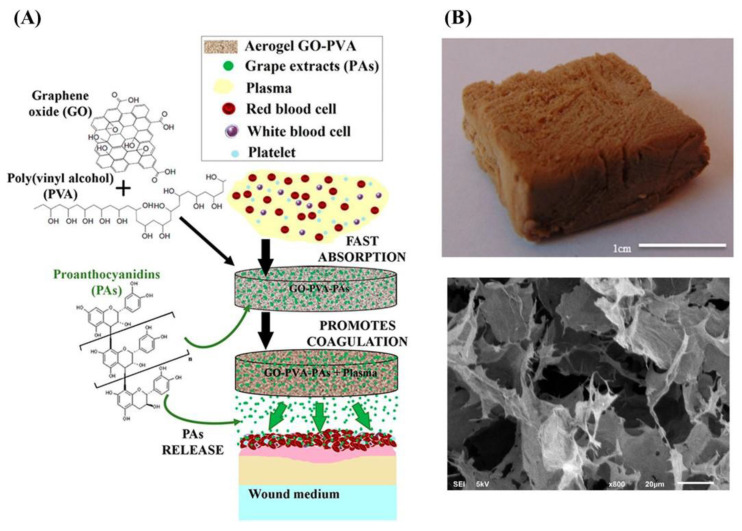
Graphical representation of GO-PVA aerogel preparation and wound-healing application (**A**). Photograph and SEM image of GO-PVA aerogel (**B**) [16].

Polyvinyl alcohol (PVA) with GO aerogels showed high structural stability and adsorption of water and blood. In the study of Fernández et al., PVA-GO aerogel prepared by freeze-drying, loaded with PAs that enhanced coagulation, was used in wound healing. The presence of PAs from grape seed (SD) and skin (SK) increased the negative charge and slowed the release, which enhanced rapid water absorption more than the unloaded aerogel (Figure 2) [16]. In a later study, the group crosslinked GO aerogels with gelatin, PVA, and chitosan (CS). Their results demonstrated that hemostatic effectiveness was enhanced not only in vitro but also in vivo compared to commercial materials. In vitro studies demonstrated that the aerogels were able to coagulate more than 60% of the contained blood and had a high level of hemocompatibility, while in vivo, the aerogels reduced the hemostasis times and blood loss significantly in comparison to the commercial materials. The aerogels were effective in accelerating hemostasis by promoting platelet adhesion, and RBCs gathered on their surface [52]. 

**Figure 3:**
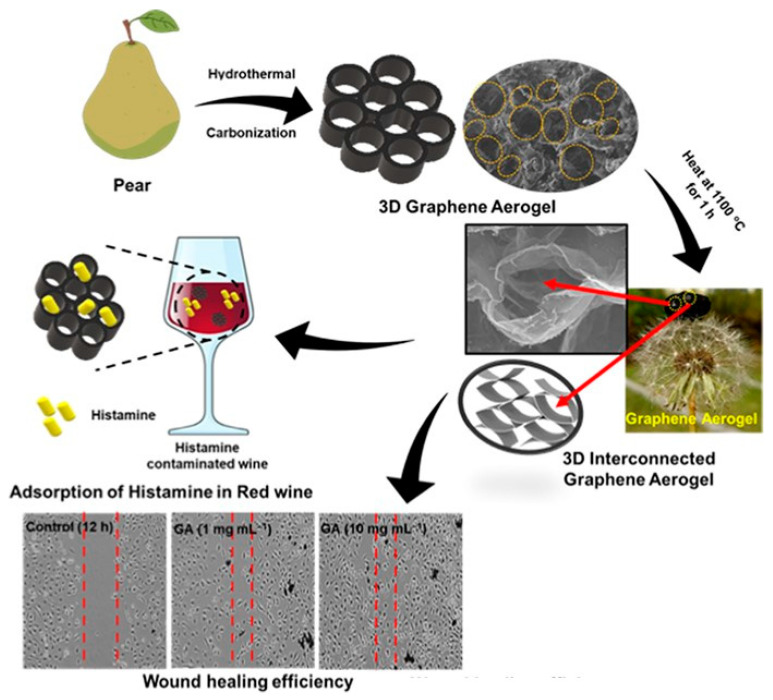
A schematic diagram illustrates the process of synthesizing GA, which is then stabilized on a dandelion head to create ultralight aerogels. GA aerogels were applied as histamine adsorbents in contaminated red wine samples and studied for potential use as wound healers [25].

The nontoxicity of GA makes it applicable in various biomedical and real-life scenarios, such as food and environment safety [25,53]. Tripathi et al. synthesized GA from *Pyrus pyrifolia* biomass that efficaciously removed histamine (HIS) from foods as an adsorbent and could be used as a wound healer. Its honeycomb-like 3D macroscopic structure, high porosity, and large internal surface area allowed it to effectively remove HIS from red wine, and it can be easily cleaned with 50% ethanol and water without losing its ability to remove HIS. Additionally, GA enhanced cell migration on scratched epithelial surface wounds (Figure 3) [25]. Similarly, GA synthesized by a one-step pyrolysis process of ammonium chloride and glucose showed excellent absorption properties for removing food toxins like various biogenic amines (BA). GA was particularly effective at eliminating three different types of BAs (such as cadaverine, histamine, and spermidine) in soy sauce. Additionally, it can completely absorb the harmful *Staphylococcus aureus* bacteria in its exponential life stage, which is a known food contaminant and can cause poor wound healing. Lung epithelial cells have also been shown to accelerate the healing rate in vitro in the presence of GA [53]. 

### 3.2. Applications of Graphene-Based Aerogels in Bilirubin Adsorption

Bilirubin is a yellow pigment that forms as a result of the breakdown of red blood cells, which binds with albumin and is transferred to the liver for removal [69]. An excessive amount of bilirubin in our body can cause hyperbilirubinemia, a condition that can lead to yellow discoloration of the tissues and the skin, and in severe cases, it may even progress to hepatic coma or death [70]. It is, therefore, crucial to remove an excessive amount of bilirubin from the blood of liver-failure patients to allow sufficient time for the impaired liver to recover or for a liver transplant. Recent research has found that graphene aerogels are an excellent bilirubin adsorbent.

A new type of bilirubin absorbent using an aerogel containing chitin/graphene oxide (Ch/GO) was synthesized in a NaOH/urea aqueous solution. The Ch/GO aerogel beads are synthesized by binding GO to the chitin matrix through hydrogen bonding and electrostatic interactions, resulting in improved thermal stability, mechanical strength, and surface area. The group absorption tests showed that the Ch/GO aerogel beads have substantial bilirubin absorption capacity and a short absorption equilibrium time under optimized conditions. Furthermore, the aerogel beads had lower hemolysis ability and improved anticoagulant properties. Due to their good mechanical strength, high surface area, better absorption properties, and blood compatibility, the Ch/GO aerogel beads are expected to be used in hemoperfusion therapy for removing excessive bilirubin [54]. Similarly, high bilirubin adsorption capacity (458.9 mg g^−1^) has been shown in chitosan/ reduced graphene oxide (CS/rGO) composite aerogel. The aerogel showed good blood compatibility with a low hemolysis ratio and negligible anticoagulant activity [55].

**Figure 4:**
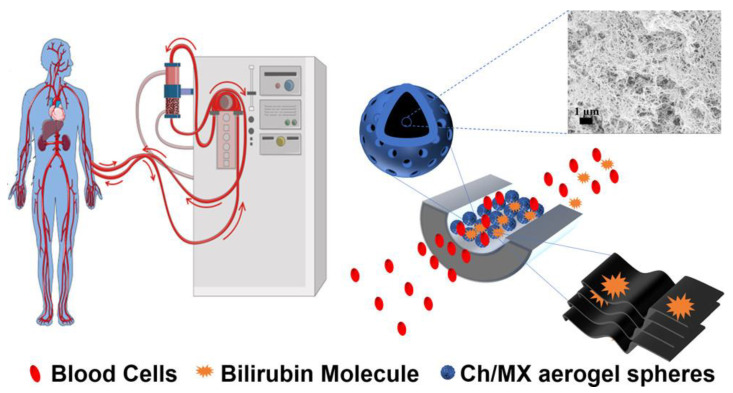
Schematic diagram of bilirubin adsorption from human blood using chitin/MXene (Ch/MX) composite aerogel [56].

MXenes are a type of 2D material where M is an early d-block transition material, and X can be nitrogen or carbon. They share similar advantageous properties with graphene, such as good electric conductivity and suitable volumetric capacitance [71]. They also possess good mechanical and adsorption properties. Therefore, Lu and colleagues created composite aerogel spheres made of chitin and MXene (Ch/MX) using supercritical CO_2_ technology. MX and chitin fibers adhere to each other through hydrogen bonding and electrostatic interactions. This resulted in aerogel spheres with improved mechanical strength, larger surface area, and various pore sizes, including mesopores and micropores. The Ch/MX aerogel spheres have a 33% shorter equilibrium time for bilirubin adsorption and a 40% increase in adsorption capacity compared to chitin aerogel spheres. Also, CH/MX aerogel spheres are highly adsorbent, averaging 142.86 mg/g in adsorption capacity (Figure 4) [56]. In a later study, the researchers added poly (L-arginine) (PLA) to Ch/MX, which absorbed up to 596.31 mg/g of bilirubin. PLA had better mechanical properties than Ch/MX and could bear up to 50,000 times its own weight. In hemoperfusion tests, Ch/MX/PLA showed higher absorption capacity compared to Ch/MX. The Ch/MX/PLA demonstrated high anti-interference properties and specific adsorption during binary and ternary competitive absorption tests [57].

### 3.3. Applications of Graphene-Based Aerogels in Drug Delivery

GA has garnered attention in recent years for its potential in drug delivery owing to its distinctive characteristics, such as high porosity, extensive surface area, and biocompatibility. The large surface area of graphene aerogels allows ample space for drug molecules to adhere or be encapsulated. Additionally, the high porosity of GA enables control of the release of drugs and keeps them consistent. The drug molecules can be released slowly over time as the aerogel gradually degrades or is subjected to specific environmental conditions, such as changes in pH or temperature. 

**Figure 5:**
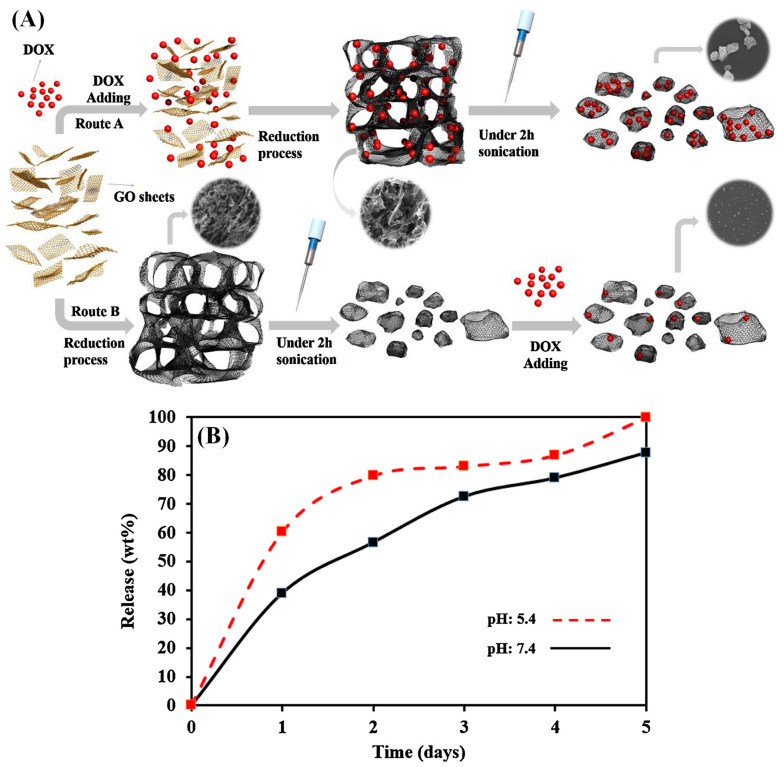
Schematic representation for the fabrication of GA and DOX loading (**A**). In vitro DOX release profile at (neutral (pH~7.4) and acidic (pH~5.4)) for 5 days (**B**) [58].

Atyabi et al. conducted a study to prepare graphene aerogel nanoparticles (GANP) loaded with anticancer drugs that exhibit pH-sensitive release. To create the GANP, GO sheets were reduced and combined with vitamin C at 40 °C, then sonicated. Non-ionized drugs, such as paclitaxel (PTX), and various ionized drugs, such as amikacin sulfate, doxorubicin hydrochloride (DOX), and D-glucosamine hydrochloride, were loaded onto the GANP. The result showed that DOX can be loaded more than PTX, indicating that the ionic characteristics and positive partial charges increase the loading capacity. D-glucosamine hydrochloride and amikacin sulfate also showed higher loading capacity than PTX, implying that aromatic structures’ π–π interactions play a critical role in drug loading. Overall, the electrostatic interactions between GO and ionized drugs were dominant conditions. Furthermore, DOX-loaded GANPs had a high pH-sensitive release rate after 5 days, making them a promising candidate for use in various organs with acidic media, such as tumors and the stomach (Figure 5) [58]. Another pH-responsive drug-carrying aerogel was proposed by Namazi et al. They proposed a pH-responsive drug-carrying aerogel made of biopolymer aerogel microspheres consisting of K-carrageenan, sodium alginate, and reduced graphene oxide (SA/K-CG/rGO). The microspheres were synthesized by using the sol–gel technique and crosslinked with a divalent cation (Ca^2+^), followed by supercritical drying. The aerogel immobilized up to 94% of the drug, amoxicillin. The release of amoxicillin from the SA/K-CG/rGO aerogel was pH-sensitive, owing to the pH-sensitive polysaccharides of SA and K-CG. At pH 5.5, amoxicillin released only 26%, a lower value than the 34% at a neutral pH 7.4 after 95 h. The release of amoxicillin was controlled by Fickian diffusion, as shown by the Korsmeyer–Peppas and Higuchi models. The minimum inhibitory concentration test results showed strong antibacterial activity for both hybrid aerogels in the presence and absence of Amoxicillin, and they were found to be nontoxic. These results suggest that the SA/K-CG/Ca^2+^-k-rGO hybrid aerogel has the potential to be an ideal carrier system for drug loading and delivery [59]. 

pH-responsive drug carriers offer a new approach to creating stimuli-sensitive and consistent drug release. The pH level strongly affects the release of 5-fluorouracil (5-FU) from the GO/hydroxypropyl cellulose/chitosan (GO/HPC/CS) aerogel. The release of 5-FU becomes more rapid and more complete under pH 5.0 due to the $NH_{2}$ groups in the aerogel, which indicates that it is beneficial for targeted acidic tumor regions. These spherical and micro-sized aerogels improved 5-FU loading and encapsulation efficiency with the GO content. Moreover, the initial burst of drug release was significantly suppressed with the increase in GO content. Additionally, the aerogel showed the consistent release of 5-FU [72]. Similarly, chitosan, carboxymethyl cellulose, and graphene oxide (CS/CMC/Ca^2+^/GO) aerogel showed sustained release of 5-FU due to π−π stacking and hydrogen bonding between GO nanosheets and 5-FU [73].

The structure of GA can be effectively controlled by hot-air drying time. GA is synthesized through a hydrothermal reaction and controlled drying process and dehydrated through freeze-drying, resulting in an interconnected three-dimensional porous network with combinations of macropores and mesopores. The porous structural properties were regulated by controlling the hot-air drying time. As a result of hot-air drying, surface area, mesopore size, bulk density, and macropore size were linearly correlated. It was finely controlled the hydrophobic (ibuprofen) and hydrophilic (diclofenac sodium) drugs released by the hot-air drying time. A second advantage of GA was its biocompatibility for local drug release. This study suggests that the hot-air drying time can effectively regulate the porous structure of GA and precisely control the drug release [60].

### 3.4. Applications of Graphene-Based Aerogels in Bone Regeneration

Developing biomaterials that mimic the natural environments of osteoblasts and osteocytes and serve as functional substitutes for bone regeneration is becoming increasingly important in reconstructive surgery. GA is a popular choice for synthetic bone grafts due to its high biocompatibility, porosity, low density, specific surface area, and mechanical strength, which can replicate natural bone’s porous, flexible, resistant, and lightweight structure [15]. Moreover, GA’s interconnected pores allow for the transportation of nutrients and chemicals to the interior of the scaffold, promoting cellular growth, vascularization, and waste disposal. Several studies have demonstrated the potential of GA in bone tissue engineering. 

**Figure 6:**
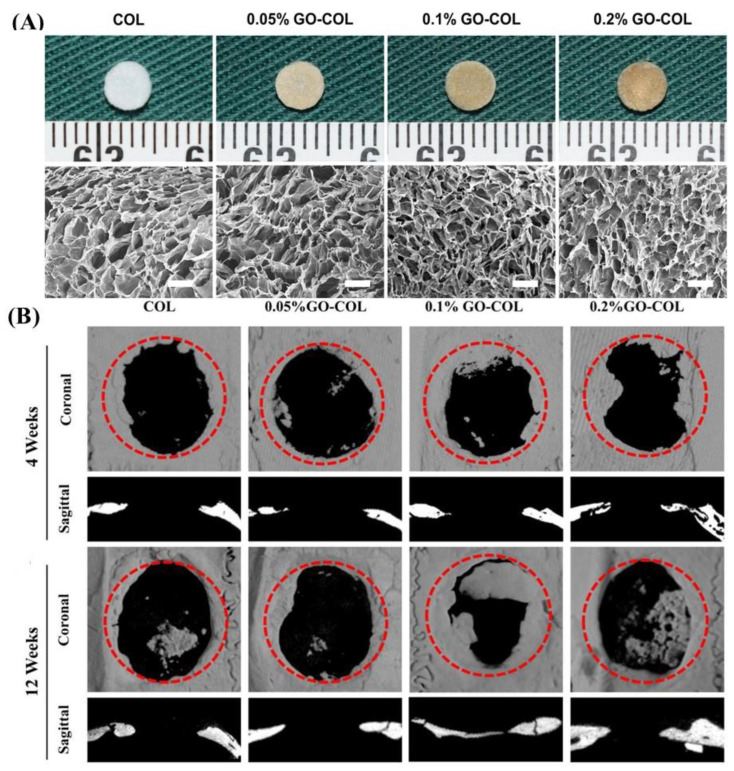
Macroscopic and SEM images of the COL and 0.05/0.1/0.2 wt% GO–COL aerogel (**A**). Micro–CT imaging analysis of aerogels implanted into rat cranial bone defects. Coronal and sagittal CT reconstruction images 8 weeks and 12 weeks post-implantation (**B**). Scale bars in A: 100 μm [24].

Hybrid aerogel consisting of GO and type I collagen (COL) synthesized by Xu et al. through the sol–gel process was an effective bone graft substitute for large bone repair. The GO-COL aerogel had high porosity and hydrophilicity. The compressive modulus of the GO-COL aerogel was improved with the GO content. In vitro, the 0.1% GO-COL aerogel showed a better biomineralization rate and cell compatibility than the 0, 0.05, and 0.2%. Moreover, it repaired bone in rat cranial defect models in vivo. The 0.1% GO-COL had good biocompatibility and osteogenic ability, which indicated it could be an effective biocompatible scaffold for bone regeneration (Figure 6) [24]. A chitosan composite aerogel with GO mimicked the bone lamellae structure. The anisotropic scaffolds played a significant role in the alignment of MC3T3-E1 cells along the longitudinal direction. The aerogel’s compressive strength and protein adsorption increased with the GO content (1–5 wt%) [74]. Incorporating GO in the scaffold improved the mechanical properties and pore formation and enhanced the bioactivity for tissue engineering. The chitosan/GO 3 wt% porous composite exhibited preosteoblast biocompatibility, making it a promising candidate for osteogenesis studies and bone repair approaches [75]. Furthermore, a study demonstrated that a poly (vinyl alcohol) (PVA) biocomposite containing chitosan and GO resulted in the formation of hydroxyapatite (HA) crystals on the surface and pores. The size of the individual crystallite was found to be dependent on the amount of GO used. The carboxylic groups on the GO and chitosan surfaces of the biocomposite scaffolds facilitated the formation of HA crystals on their surface, resulting in nucleation [76]. The reduced graphene oxide aerogels (A-RGO), which were functionalized with chitosan, acted as a soft interfacial template on the surface of A-RGO, accelerating hydroxyapatite particles’ mineralization and growth. Soft templating A-RGO with chitosan showed a higher mineralization rate, bioactivity, and cell differentiation when compared to bare RGO. Furthermore, the mineralized samples showed a higher cell viability rate, osteogenic differentiation, and matrix formation, indicating their potential for in situ bone regeneration applications [77]. In a study conducted by Mishra et al., it was found that nanocomposite biocompatible scaffolds made with GO and other materials such as chitosan, gelatin, and nanobioglass showed potential for restoring bone tissue. The incorporation of GO nanoparticles increased the mechanical strength of the scaffold. However, it was observed that higher concentrations of GO nanoparticles led to an increased degradation rate compared to the positive control. The Ch-G-NBG-GO scaffolds were also found to improve the osteogenic differentiation of the MG-63 cells. These findings suggest that the Ch-G-NBG-GO scaffolds have a promising potential for bone tissue engineering applications [78].

Researchers have experimented with using polydimethylsiloxane (PDMS) to support cell growth and implant surface proliferation to improve osseointegration in cell-based implants. PDMS is known for its high oxygen solubility, which makes it an effective material for this purpose. Wallace et al. created a PDMS-based 3D structure coated with reduced graphene oxide (RGO), which is both porous and cytocompatible. This scaffold has diverse pore sizes ranging from 10 to 600 μm and is durable with good mechanical strength. The scaffold supports the growth and differentiation of human adipose stem cells (ADSCs) to an osteogenic cell lineage, which is required for improved osseointegration in orthopedic surgery [79]. Poly (2-hydroxyethyl methacrylate) (PHEMA) is another synthetic polymer broadly used in bone tissue engineering. A porous composite scaffold of PHEMA/GO/gelatin developed by Salehnia et al. showed osteogenic potential. It had a porous interconnected structure with a pore size of 50~300 μm, appropriate for bone regeneration. As the GO contents increased, the compressive modulus of the scaffold also increased. Its electrical conductivity was in the semiconductor range, and its degradation rate seemed to align with the natural progression of bone repair. It had no toxicity against human bone-marrow-derived mesenchymal stem cells (hMB-MSCs). Additionally, PHEMA/GO/gelatin scaffolds could effectively induce the cell differentiation of hMB-MSCs [80]. A 3D porous scaffold of nanosized graphene oxide (nGO) with starch also exhibited potential for bone regeneration. The nGO and starch were attached by an esterification reaction to prepare functionalized starch (SNGO). The scaffold showed good biocompatibility to MG63 cells. Moreover, the scaffold was the effective anchoring site for inducing CaP recrystallization in simulated body fluid [81]. 

### 3.5. Applications of Graphene-Based Aerogels in Biosensors

GA has gained significant interest in biosensors due to its excellent electrical conductivity and large surface area. A quasistatic or low-frequency dynamic strain or pressure sensor is typically suitable for these applications.

A study led by Shen created a hybrid aerogel made of MXene and rGO that can detect low pressures such as human heartbeats or finger movements. The synthesized aerogel creates a flexible pressure sensor with a broad detection range of 0 to 40 kPa, fast response time, and cycling stability. The aerogel prepared via the freeze-drying method exhibits an interconnected three-dimensional network, hierarchical porous architecture, excellent mechanical properties, and flexibility [82]. Another pressure sensor has been developed using bacteria cellulose and caffeic acid-rGO composite aerogel. Unlike existing petrochemical flexible sensors, the sensor was created using a green and easy process, which helps to avoid potential damage to human health. The pressure-sensitive aerogel sensor had a unique three-dimensional hierarchical porous structure, which made it highly sensitive with an exceptionally low detection limit and fast response time. It also exhibited excellent reproducibility, as confirmed by 1000 cycles of pressure loading and unloading. Based on these properties, researchers believe that pressure-sensitive aerogel sensors can be used in healthcare devices and wearable electronics to monitor various physical movements, such as vocal cord vibrations, facial expressions, and real-time recording of joint movements [61]. Similarly, a study found that sensors made from polyimide (PI) and rGO aerogel using bidirectional freezing techniques have excellent pressure sensitivity. These sensors can detect even the slightest changes in pressure with an ultralow detection limit. The sensors have a broad detection range and a fast response time, which make them ideal for use in high-performance wearable electronic devices and flexible pressure sensors. These sensors can also function well in harsh environments, showing stable piezoresistive performance even at extreme temperatures ranging from −50 °C to 200 °C in air. Therefore, they can detect overall human motions, including small- and large-scale movement monitoring [83].

**Figure 7:**
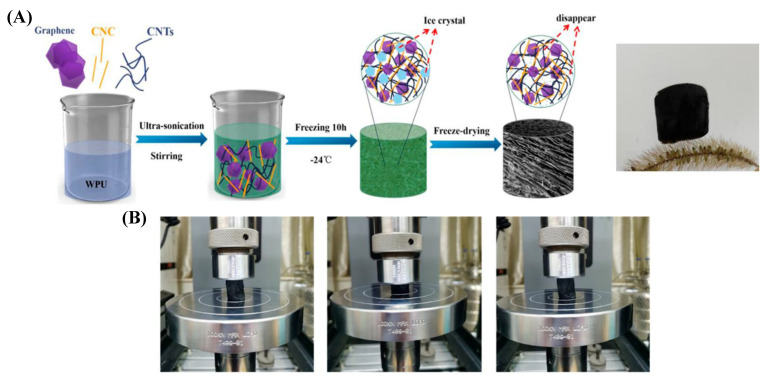
Schematic illustration of the fabrication process for the CNTs/graphene/WC composite aerogel, along with a digital photo of the final product (**A**). The compressing and releasing process of the CNTs/graphene/ WPU/CNC is shown in photographs (**B**) [62].

An aerogel made from carbon nanotubes (CNTs), graphene, waterborne polyurethane (WPU), and cellulose nanocrystal (CNC) has been prepared by using a simple solution-mixing and freeze-drying technique. The aerogel was highly porous, mechanically robust, and exhibited excellent sensitivity and stability and an ultra-low detection limit, making it ideal for high-performance pressure sensors. These piezoresistive sensors can detect various human motions and show excellent thermal insulation properties, enabling them to withstand high temperatures of up to 160 °C without damage. Due to these features, the CNTs/graphene/WPU/CNC aerogels are perfect for use in flexible, wearable electronics (Figure 7) [62].

The production of piezoresistive sensors using GA has been a challenge due to the irregular microstructure of most isotropic GAs, which leads to limited linearity and poor stability. Therefore, Jiang et al. recently fabricated an extensive piezoresistive sensor using an aerogel including methyl-cellulose-reinforced rGO (MC/rGO). The process involved mixing, directional freezing, freeze-drying, and steaming. The chemical bonding and intermolecular forces between MC and GA were controlled by steaming reduction at 120 °C. Most GA is damaged by high-temperature treatment. However, this piezoresistive sensor has wrinkled lamellae with hierarchical pores, which are advantageous in minimizing stress concentrations. The combined structure of reinforced and wrinkled lamellae with hierarchical pores provides MC/GA with superior linearity in a much more comprehensive range, high sensitivity, a rapid response, and dependable cyclic performance. MC/GA sensors may be promising in various motion-detecting fields and health monitoring [63]. Similarly, a composite of crosslinked graphene nanosheets and PVA aerogel can be used as a wearable device for motion detection. The aerogel exhibits superior properties such as remarkable resilience and compressibility, outstanding electromechanical sensing performances, excellent durability and mechanical strength, and satisfactory fire resistance. It can operate at an ultralow voltage and efficiently respond to various strains and pressures even at temperatures exceeding 300 °C. The aerogel also has ultra-high-pressure sensitivity, remarkable sensing stability, and durability and can detect vibration signals with extremely high frequencies. These biomimetic crosslinked GA can be used not only as wearable devices for monitoring human movements but also for nondestructive detecting of cracks in engineering structures [84].

The GO nanosheets hybridization with functionalized carbon fibers (CF) resulted in a high-performance multifunctional hybrid carbon aerogel. The aerogel had many unique properties, including low density, high electrical conductivity, low thermal conductivity, remarkable strain–electrical responses, and outstanding electrochemical energy storage performance. When compared to carbon microfibers (CMFs), aerogels containing carbon nanofibers (CNFs) exhibited better mechanical strength due to their improved interfacial adhesion. CNFs also provided numerous electron transport paths, resulting in decreased electrical resistance and enhanced thermal conductivity. The hybrid GAs maintained their elasticity through at least 100 cycles at a 50% strain and had a compressive strength of 56.7 kPa [85]. Another pressure sensor, made of a high-performance flexible dielectric nanocomposite (PCGA), has been developed. In order to reach the percolation threshold with a small amount of rGO, the polydimethylsiloxane (PDMS) matrix was backfilled into a prefabricated chitosan-reduced graphene oxide (CGO) aerogel, which possessed a three-dimensional conductive network. This procedure greatly improved its dielectric constant. The PCGA composite exhibits a favorable combination of excellent sensitivity and a wide sensing range. It can efficiently monitor numerous human actions in real time. This novel composite material holds promise for applications in artificial electronic skins and wearable healthcare devices [86].

**Figure 8:**
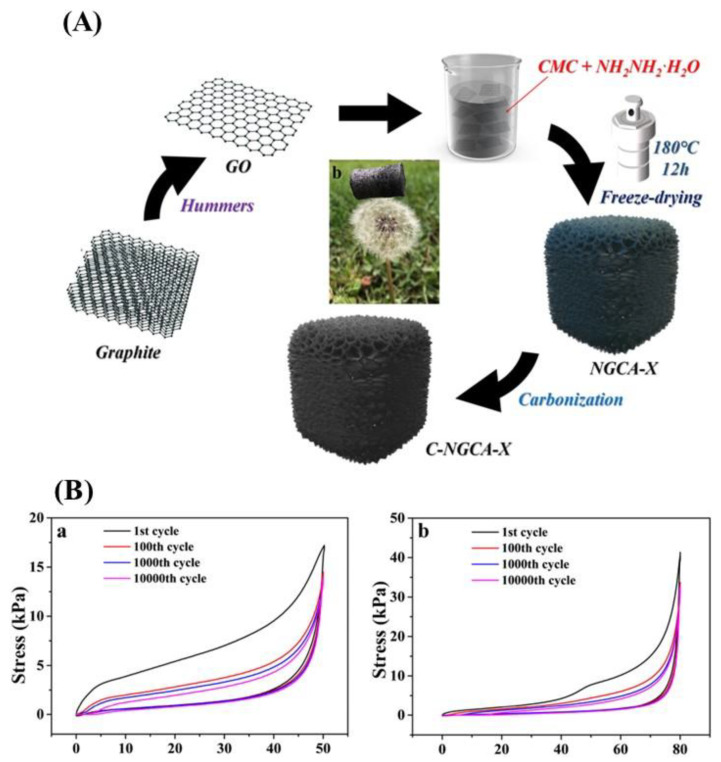
A schematic illustration shows how NGCA-X and C-NGCA-X aerogels were made (**A**). NGCA-10 corresponds to 10,000 compression cycles at (**a**) 50% strain and (**b**) 80% strain (**B**) [65].

A new type of aerogel made from a composite of nitrogen-doped graphene, which is exceptionally robust, elastic, and lightweight, has been developed. The aerogel is created by combining GO, dopamine, and polyaniline (PANI) dispersions and then chemically reducing, crosslinking, freeze-drying, and thermally annealing the mixture. This process results in an aerogel with excellent stability under compression. Adding dopamine to the graphene sheet enhances its toughness, while the π–π stacking interaction between PANI and the graphene sheet strengthens the overall structure of the aerogel. The GDPA composite aerogel has excellent compressibility, toughness, and mechanical strength, making it highly suitable for healthcare detection, wearable electronics, and intelligent packaging. It also has a lower detection limit and a more comprehensive detection range, adding to its potential [64]. GO nanosheets were assembled into a highly porous hydrogel through alkali induction to develop an ultralight graphene aerogel. During the process, the added reductant, formamidine sulfonic acid (FAS), eliminated the oxygen-containing functional groups from the hydrogel. The hydrogel was quickly dehydrated at a high temperature within 30 min to produce pure aerogel of FGA. After additional calcination, an ordered structure aerogel of AFGA was obtained. This aerogel has ultralow density, high conductivity, supercompression recovery performance, high sensitivity, large reversible compression cycle performance, and high fatigue resistance, which makes it an excellent candidate for various sensing fields such as monitoring personal health and detecting pressure distribution. It has great potential in exploring the application of aerogels in sensors [87]. A simple and efficient hydrothermal self-assembly method was utilized to create an NGCA-X aerogel with varying ratios of rGO to carboxymethyl cellulose (CMC) (NGCA-X). The NGCA-X aerogel was then carbonized to synthesize a rGO aerogel, which exhibited a multi-scale hierarchical cellular structure. It imitated the intricate design of deep-sea glass sponges (Figure 8A). The rGO aerogel had a hierarchical cellular structure that spanned from the nano- to the macro-scale, achieved by transforming the carbon wall into a cellular structure. The aerogel was highly flexible and tough, even under extra-high compressions. Its compressive strength was outstanding, overcoming the limitation of low-stress tolerance observed in typical graphene aerogels. Moreover, it exhibited a high level of electrical conductivity and an extremely stable current signal response, even after tens of thousands of compression cycles under extreme strain (Figure 8B). These outstanding properties of the rGO aerogel make it a promising candidate for use as a piezoresistive sensor, showing excellent stability and a broad detection range [65].

## 4. Conclusions and Future Perspectives

Aerogels are three-dimensional, ultra-light, solid networks with high porosity and large specific surface areas. Combined with graphene, aerogels improve electrical conductivity, mechanical strength, surface area, and adsorption capacity. GA is a fascinating class of materials with a diverse range of potential applications, particularly within biomedicine. In this review, we have discussed its biomedical applications, including wound healing, hemostasis, bilirubin adsorption, drug delivery, bone regeneration, and biosensors. The antibacterial properties of GAs and their ability to promote cell growth make them ideal materials for wound-healing applications. Future studies should focus more on the antibacterial activity of GAs to increase their antibacterial activity. Additionally, GAs show significant potential in treating traumatic bleeding and can be used as a hemostatic agent. These aerogels quickly adsorb plasma and accelerate coagulation due to the presence of GO. However, the drawback of GO aerogels is that they cannot stimulate hemocytes to control bleeding when they come into contact with blood [88]. The large surface area and porous structure of GAs make them an ideal platform for drug delivery systems. They allow the controlled release of drugs or therapeutic agents. Future research can focus on optimizing these systems for cancer treatment and regenerative medicine [58]. Moreover, GAs can also be functionalized with specific bioactive molecules to detect various biomarkers, pathogens, or toxins in biological samples [89]. 

GA has the potential to be used as a scaffold for tissue engineering. Its mechanical strength and biocompatibility make it suitable for creating structures that mimic the extracellular matrix of various tissues. GA has shown promising mechanical properties for bone regeneration. However, there is a concern regarding the inflammatory response and the formation of fibrous tissue, which can be challenging to address. Other obstacles associated with GA include concerns over toxicity and biodegradability, making it imperative to investigate its biocompatibility and biodegradability in vivo. Clinical trials are necessary to establish the safety and effectiveness of GA in real-world medical applications. GA is a highly responsive material that is sensitive to changes in its environment. This characteristic makes it a great option for use in biosensors. Wearable biosensors are becoming increasingly popular for continuous health monitoring and point-of-care diagnostic tools. In the future, we may see GA being used for disease detection as well. We believe the advancements in designing the pore shape, size, and electrical stimulation of GA constructs improved their performance.

## Figures and Tables

**Table 1:** Available control variables and their impacts on the pore size in the sol–gel method.

Controlled Factor	Effect on Pore Size	References
Precursor concentration	The higher the graphene concentration, the smaller the pores	[29]
pH	The lower the pH, the smaller the pores	[30,31]
Surfactant	Depends on the surfactant used	[32]
Resorcinol–formaldehyde	The less the resorcinol–formaldehyde, the smaller the pores	[33]

**Table 2:** Available control variables and their impacts on pore size in the freeze casting.

Controlled Factor	Effect on Pore Size	Reference
Temperature	The lower the freezing temperature, the smaller the pores	[35]
Precursor concentration	The higher the precursor concentration, the smaller the pores	[38]
Flake size	The larger the graphene flakes, the smaller the pores	[39]
Suspension viscosity	The higher the viscosity, the smaller the pores	[39]

**Table 3:** Available controlled factors and their impacts on pore size in hydrothermal reduction.

Controlled Factor	Effect on Pore Size	Reference
Temperature	The higher the temperature, the smaller the pores	[44]
pH	The higher the pH, the larger the pores	[28]
GO dispersion	The more π−π interactions between GO sheets in the primary solution, the larger the pores	[45]
Reducing agent	Depends on reducing the agent	[46]
Post-treatment	If annealing the aerogel, the pore size decreases	[47]
Time	The longer the hydrothermal reduction time, the smaller the pores	[48]

**Table 4:** GA for biomedical applications: the compositions and physical properties.

Types of GA	Fabrication Method	Pore Size/Volume	Electrical Conductivity /Resistivity	Surface Area	Application	Ref
GO/G	Physical interaction (GO oxygenated groups and G amine groups)	Basic aerogels: 42.17 ± 12.54 μm Acid aerogels: 25.30 ± 10.38 μm	-	-	Hemostatic /Wound healing	[49]
G/GO G/GO/PA (5%) G/GO/PA (10%)	Microwave-assisted synthesis	25.0 ± 1.9 µm 24.9 ± 1.6 µm 24.7 ± 1.8 µm	-	-	Hemostatic /Wound healing	[50]
GO/PEG/PA		20.5 ± 6.3 µm	-	-	Hemostatic	[51]
GO/CS GO/GEL GO/PVA	Microwave-assisted synthesis	32.4~36.8 μm	-	-	Hemostatic	[52]
GA from *Pyrus pyrifolia* biomass	Hydrothermal and post-pyrolysis process	0.27 cm^3^/g	-	$\sim 480\;m^{2}/g$	Wound healing	[25]
GA	One-step pyrolysis of glucose and ammonium chloride	2.24 cm^3^/g	-	$\sim 2860\;m^{2}/g$	Wound healing	[53]
Ch/GO-5 Ch/GO-10 Ch/GO-20	Using hybrid aqueous solution system of NaOH and urea blending with chitin and GO using H_2_SO_4_ as a coagulation bath	0.8307~1.0270 cm^3^/g	-	159.81~186.98 m^2^/g	Bilirubin adsorption	[54]
CS/rGO	Chemical reduction method	0.076~0.12 cm^3^/g	-	27.05~48.88 m^2^/g	Bilirubin adsorption	[55]
Ch/MX aerogel sphere	Supercritical $CO_{2}$ technology	0~37 nm	-	123~218 m^2^/g	Bilirubin adsorption	[56]
Ch/MX/PLA	Supercritical $CO_{2}$ technology		-	107~153 m^2^/g	Bilirubin adsorption	[57]
GA NPs	Reduction/aggregation of GO sheets in the presence of vitamin C	30∼300 μm	-	-	Drug delivery	[58]
SA/K-CG/rGO	Sol–gel technique	1~6 nm	-	204.90 m^2^/g	Drug delivery	[59]
Gn aerogel	Hydrothermal reaction and controlled drying process	30~500 μm	-	88.3~147.7 m^2^/g	Drug delivery	[60]
GO/COL	Sol–gel process	100~160 μm	-	-	Bone regeneration	[24]
Bacteria cellulose /rGO	Reduction, mixing, and freeze-drying technique	40~300 μm	-	-	Biosensors	[61]
CNTs/Gn/WPU /CNC	Facile solution mixing and freeze-drying technique	110~180 μm	-	-	Biosensors	[62]
MC/GA	Stirring, freeze-drying, steaming	-	Resistance normal to the lamellae: 158 kΩ at thickness of 2.0 mm, parallel to the lamellae: 394 kΩ at a length of 10.0 mm	-	Biosensors	[63]
GO/DA/PANI combined nitrogen-doped aerogel	Crosslinking at 90 °C, freeze-drying, thermal annealing	-	Without doping: 11.0 S/m GDA: 16.9 S/m GPA: 82.3 S/m GDPA: 56.7 S/m	-	Biosensors	[64]
NGCA-X aerogels	Facile hydrothermal self-assembling strategy	-	C-NGCA-10: 42.7 S/m	-	Biosensors	[65]

## Data Availability

Not applicable.
